# *in silico* Surveillance: evaluating outbreak detection with simulation models

**DOI:** 10.1186/1472-6947-13-12

**Published:** 2013-01-23

**Authors:** Bryan Lewis, Stephen Eubank, Allyson M Abrams, Ken Kleinman

**Affiliations:** 1 Social & Decision Informatics Laboratory, Virginia Tech Research Center, 900 N. Glebe Road, Arlington, VA, 22203, USA; 2 Network Dynamics and Simulation Science Laboratory, Virginia Bioinformatics Institute (0477), Virginia Tech, Blacksburg, VA, 24061, USA; 3 Department of Population Medicine, Harvard School of Medicine, 133 Brookline Ave, Boston, MA, 22201, USA

**Keywords:** Surveillance, Simulation, Outbreak detection, Evaluation, Agent-based model, Influenza-like illness

## Abstract

**Background:**

Detecting outbreaks is a crucial task for public health officials, yet gaps remain in the systematic evaluation of outbreak detection protocols. The authors’ objectives were to design, implement, and test a flexible methodology for generating detailed synthetic surveillance data that provides realistic geographical and temporal clustering of cases and use to evaluate outbreak detection protocols.

**Methods:**

A detailed representation of the Boston area was constructed, based on data about individuals, locations, and activity patterns. Influenza-like illness (ILI) transmission was simulated, producing 100 years of *in silico* ILI data. Six different surveillance systems were designed and developed using gathered cases from the simulated disease data. Performance was measured by inserting test outbreaks into the surveillance streams and analyzing the likelihood and timeliness of detection.

**Results:**

Detection of outbreaks varied from 21% to 95%. Increased coverage did not linearly improve detection probability for all surveillance systems. Relaxing the decision threshold for signaling outbreaks greatly increased false-positives, improved outbreak detection slightly, and led to earlier outbreak detection.

**Conclusions:**

Geographical distribution can be more important than coverage level. Detailed simulations of infectious disease transmission can be configured to represent nearly any conceivable scenario. They are a powerful tool for evaluating the performance of surveillance systems and methods used for outbreak detection.

## Background

Detecting outbreaks is a crucial task for public health officials. Distinguishing between truly significant outbreaks and normal disease incidence and to do so as early as possible is a laborious and imperfect science [1]. Techniques and methodologies have been developed to screen the increasingly large volumes of data useful to the task [2,3], yet despite this progress, gaps in performance remain. We present an agent-based simulation methodology to address these challenges.

Developing and evaluating outbreak detection is challenging for many reasons. A central difficulty is that the data used to “train” detection algorithms are unique and relatively brief historical samples and thus do not represent the full range of possible background scenarios. The same dearth of historical data complicates evaluation. In systems where only a count of cases is provided, plausible synthetic data are relatively easy to generate, and can aid in development and evaluation. When evaluating a surveillance system with more precise information, notably detailed geographic locations, simple approaches to generating hypothetical case counts are not plausible.

Increasingly realistic simulations of infectious disease spread in highly detailed synthetic populations have emerged in recent years [4-9]. Agent-based simulations can represent real-world populations and the day-to-day processes that determine disease-spread and health care seeking behavior. Combining these capabilities with detailed knowledge of a surveillance system allows for the construction of plausible synthetic surveillance data streams. These *in silico* surveillance data streams can be configured to represent nearly any conceivable set of scenarios, making them a powerful tool for studying surveillance systems and outbreak detection algorithms.

We use a novel highly detailed agent-based model, which produces realistic geo-spatial and temporal clustering of influenza-like illness (ILI), to create a library of daily zip code counts from 100 influenza seasons. From this synthetic data, we construct six different surveillance streams that include coverage patterns and health-care-seeking behaviors. For evaluation, we insert artificial outbreaks into the synthetic surveillance data streams at different locations and times and measure the performance of an outbreak detection protocol based on scan statistics. This approach is novel, and represents (to our knowledge) the first time a large-scale agent-based model has been used to evaluate a spatio-temporal outbreak detection tool.

## Methods

### Synthetic population creation

The methods used to generate a dynamic and highly detailed synthetic population have been described in detail previously [4,10]. Real-world data and behavioral models are used to generate data sets containing the location of every individual in the population at every moment in the day. Census data and household location data are used to assign individuals to households. The activity profiles, which determine locations throughout the day, were drawn from large surveys and are assigned to each individual based on household and individual demographics, ensuring that individuals perform activities and in locations appropriate to their age and household structure [11,12]. An iterative fitting process is used to constrain the distances traveled to fit distributions from the activity surveys. This proto-population is then updated with additional information specific to the context of the study being performed, e.g., zip code locations were calculated for all individuals to determine their membership in the various synthetic surveillance systems.

### Disease modeling

**Figure 1:**
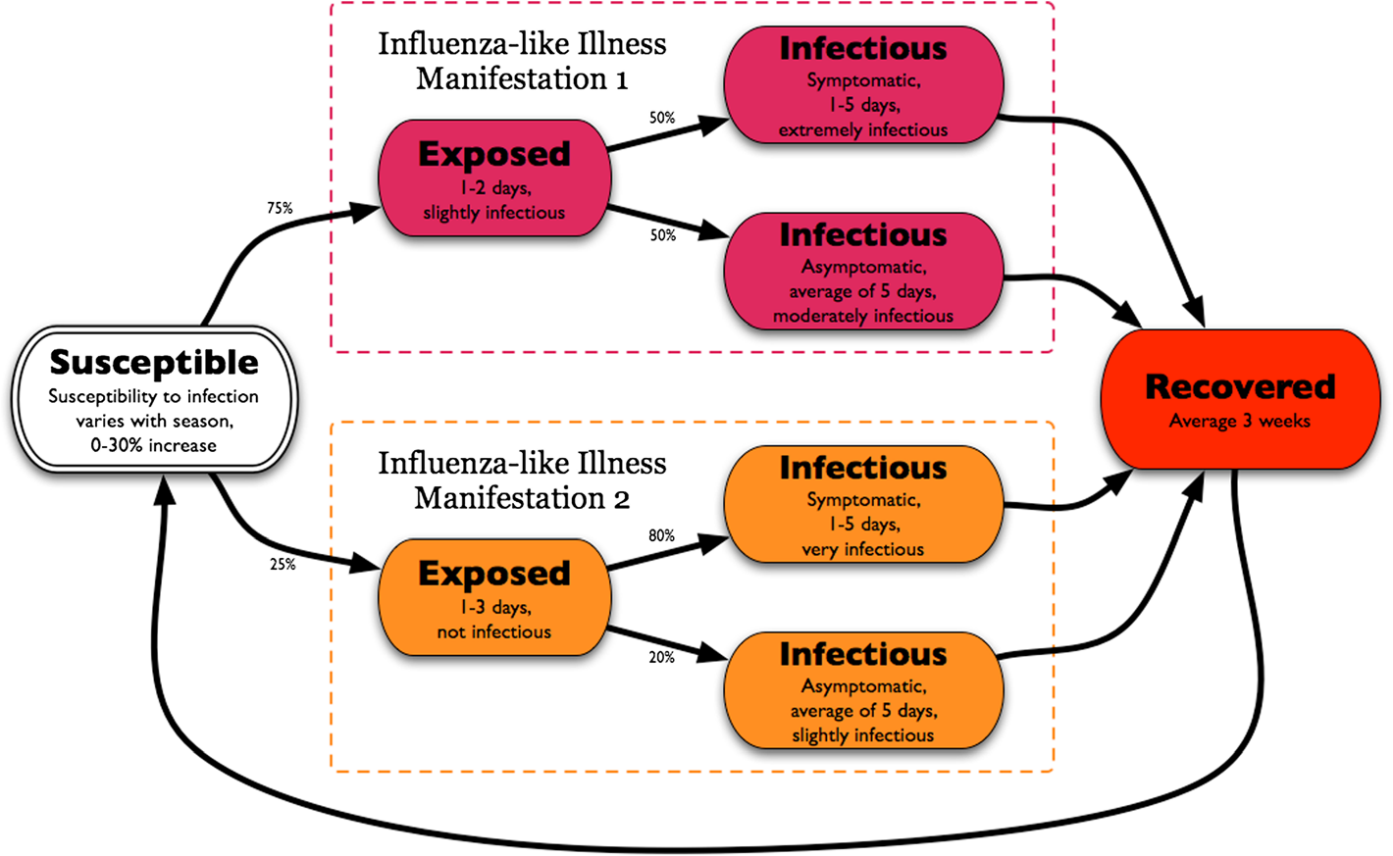
**Finite state machine representation allows for a flexible representation of a disease process.** Each state determines the duration in that state, level of symptoms, susceptibility, and infectiousness. For example, susceptible individuals have a 75% chance that upon exposure to an infectious contact and successful infection they will transition to the latent Exposed state in ILI Manifestation 1; after 1 to 2 days they will then transition to one of the more Infectious states with a 50% chance of having symptoms.

Influenza and ILI-causing pathogens are transmitted through aerosol contact routes. A discrete event simulation engine simulates the spread of these diseases through the simulated population based on contacts between uninfected and infectious individuals [13,14]. Figure 1 illustrates the structure for the ILI disease model, which is an elaboration on the classic SEIR (Susceptible, Exposed, Infectious, and Recovered) model that accommodates multiple manifestations, asymptomatic infections, and a temporary Recovered state. The detailed disease model (see Additional file 1) used in the simulation includes a number of parameters taken from the literature to represent the broad spectrum of influenza-like illnesses [15-17]. Here, the probability of transmission is a function of the duration of contact, symptom severity, type of treatments either individual may have taken, and level of susceptibility of the uninfected individual. For example, if a person, asymptomatically infectious with manifestation 1, was in contact with an individual who was uninfected but had increased susceptibility due to the point in the season, the probability of transmission would be scaled by a level of infectiousness (0.5, due to lack of symptoms), susceptibility (1.15, due to season), and not influenced by treatment (no treatment because no symptoms). Under these conditions and 12 hours of interaction between these individuals the calibrated probability of infection within the simulation would be 0.008322.

**Figure 2:**
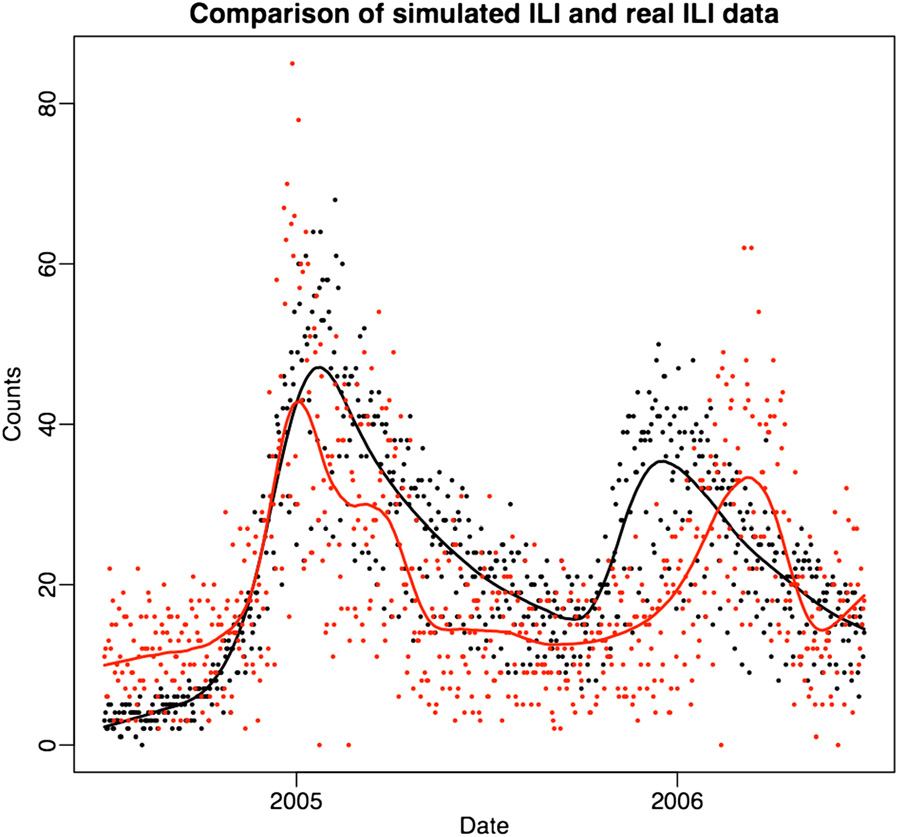
**Two years of real ILI data (red) compared to 2 years of data from a single simulation (black).** Each dot represents the total number of cases seen in the surveillance stream on a single day; the curves are a smoothed fit to these data.

The characteristic seasonal pattern of ILI infections (Figure 2) was generated. First, an ILI disease was spread in the simulated population, and calibrated to a level that maintained infection at a stable level over several years. Seasonal effects were added by gradually increasing the overall susceptibility to infection of the population in a controlled fashion. The start of this change occurred at a “season onset” date selected at random from a distribution based on historical CDC data [18]. Year-to-year variability in season onset and pattern was simulated by choosing from among ten templates inferred from the historical shapes of influenza incidence. Stochastic variability inherent in the system introduced variations between seasons such that even realizations based on the same template differ dramatically.

### Care seeking modeling

Health care seeking behavior was modeled by combining the marginal delay from symptom onset to health care seeking distribution [19] and day-of-the-week distribution calculated from Harvard Pilgrim Health Care (HPHC) clinic data for ILI. The resulting joint distribution determines the distribution of the number of days following symptom onset that an individual will wait before seeking health care, given the day of the week. For example individuals who would seek care and start experiencing symptoms on a Monday have a 37% chance of seeking care that Monday, but only an 8% chance of seeking care two days later on Wednesday; whereas if the symptoms start on a Saturday there is a 25% chance they will seek care on that Saturday and a 15% chance they will wait two days till Monday. The probability an affected symptomatic individual seeks health care, is diagnosed correctly, and is reported in the surveillance system can be adjusted to calibrate to a specific real-world surveillance system, or can be parameterized based on data. For example, the effects of a higher co-pay could be introduced to reduce the probability of seeking care, or the probability of correct diagnosis could be reduced to reflect the difficulty of diagnosis.

### Surveillance system

Six different surveillance systems were designed. These represented three different coverage levels and two different geographic distributions of its membership. The “Natural” geographic distribution is based on the naturally occurring distribution of the Harvard Pilgrim Health Care (HPHC) members, whereas, the “Uniform” distribution draws members uniformly across the same geographic areas. The proportions of HPHC members living in each zip code in the Boston metro area were determined. The 246,606 members resided in 210 of the 755 zip codes in the synthetic data region (ranging from 64 to 58,857 people in a single zip code), which is 6% of the entire synthetic population. The selection was done by household and was used as one of the synthetic surveillance systems (Natural 6). The coverage level was doubled (to ~12%) and tripled (to ~18%) while maintaining the same surveillance geography (Natural 12 and 18). The same three coverage levels were combined with a “Uniform” surveillance geography that drew members from the same 210 zip codes, but with an equal proportion of each zip code as members. Thus, the six surveillance systems evaluated consist of combinations of the two geographic distributions (Natural and Uniform) and the three coverage levels (6%, 12%, and 18%).

### Artificial outbreaks

To test the performance of the different surveillance systems, artificial outbreaks were randomly inserted into the simulated ILI background signals. Each outbreak consisted of cases inserted into the surveillance stream (not subjected to care-seeking behavior modeling) over 14 days, and follows the temporal pattern “2,0,2,0,2,2,0,4,2,0,2,2,0,2” cases per day. This temporal pattern is the same for all insertions for the sake of offering a single comparison across seasons and geographic space. These numbers were scaled with the coverage level of the surveillance system being tested (multiplied by 2 at the 12% level and by 3 at the 18% level). This artificial outbreak was meant to simulate a small concentrated outbreak of influenza-like illness and was based on experience from the Massachusetts Department of Health. The primary location for insertion was randomly chosen from all zip-codes in the population and two additional locations near the primary location were also chosen. The inserted cases were then distributed at random to these three locations.

### Outbreak detection

We assessed the ability to detect the inserted outbreaks using a space-and-time permutation scan statistic [20,21]. Cases from the synthetic population and from the artificial outbreak were assigned a geographic location based on the centroid of their home zip code. The approach finds the cluster least likely to have occurred by chance, where a cluster consists of 1–14 days in any fixed circular region, and assigns a p-value to this cluster using Monte Carlo techniques. This process is repeated daily as the data accrues. The scan implemented uses 90 days of historical data to establish “normal” patterns.

### Evaluation of outbreak detection

Outbreaks were combined with the simulated ILI background by adding the case counts from the artificial outbreak to the appropriate zip code case count on the appropriate day. The outbreak detection protocol was performed for every day in which inserted cases could have been included in a 14-day putative cluster. The scan statistic was considered to have “signaled” if the p-value fell below a given decision threshold. In this study eight different decision thresholds (p = 0.033, 0.01, 0.0075, 0.005, 0.004, 0.003, 0.002, 0.001) were evaluated.

If any scan signal with a p-value at or below a given decision threshold overlapped one of the locations of the inserted outbreak, the inserted outbreak was considered to have been detected at that decision threshold. These are true positives. After attempting to detect the outbreak, the inserted cases were removed before the next outbreak was inserted and its detection attempted. Twelve test outbreaks were inserted in each of the 100 simulated ILI seasons on randomly chosen dates.

This outbreak detection protocol was also performed for the one-year simulated ILI counts without any inserted outbreaks. Any outbreaks signaled in the simulated ILI background data were considered false positives. Any identified signals that overlapped in time and space were “collapsed” into a single “cluster” and designated a false-positive.

### Computations

The simulations of ILI disease and health care seeking behavior were performed by EpiSimdemics [14,22] on an SGI cluster with 96 compute nodes, each with 2 Intel Quad-Core Xeon E5440 processors. The SaTScan analyses were conducted on the same cluster. In total 36,500 scans (100 years * 365 days) with no inserted cases were performed, and 32,400 scans (100 years * 12 insertions * 27 days) were performed for inserted outbreak detection. These simulations and analyses used 84,000 compute hours and required 187 GBs of disk space. Outbreak detection analysis was conducted using SaTScan version 7.0.3 (from http://www.satscan.org) and R version 2.9.0 (from r-project.org). The analysis package, including code to automate the SaTScan outbreak detection and analysis, will be made available as an R package.

## Results and discussion

Much as any single influenza season is not the same as previous influenza seasons, the simulated ILI surveillance counts are not the same as the real-world ILI surveillance counts, however they share similar characteristics. Figure 2 shows both real data from HPHC and simulated data. ILI surveillance data is characterized by strong seasonal influence and a day of the week pattern, which are evident in the Figure.

The surveillance of the simulated ILI counts without any inserted outbreaks informs decisions about the real-world tenability of a surveillance system. Typically overburdened public health authorities cannot support systems with too many false positives. We found 244 overlapping “clusters” of signals in the 100 years of data in the “Natural 6” system at the 0.001 decision threshold, when the expected number under the null is 36.5. We also saw 547, 669, 394, 626, and 732 in the Natural 12, Natural 18, Uniform 6, 12, and 18 systems respectively.

**Figure 3:**
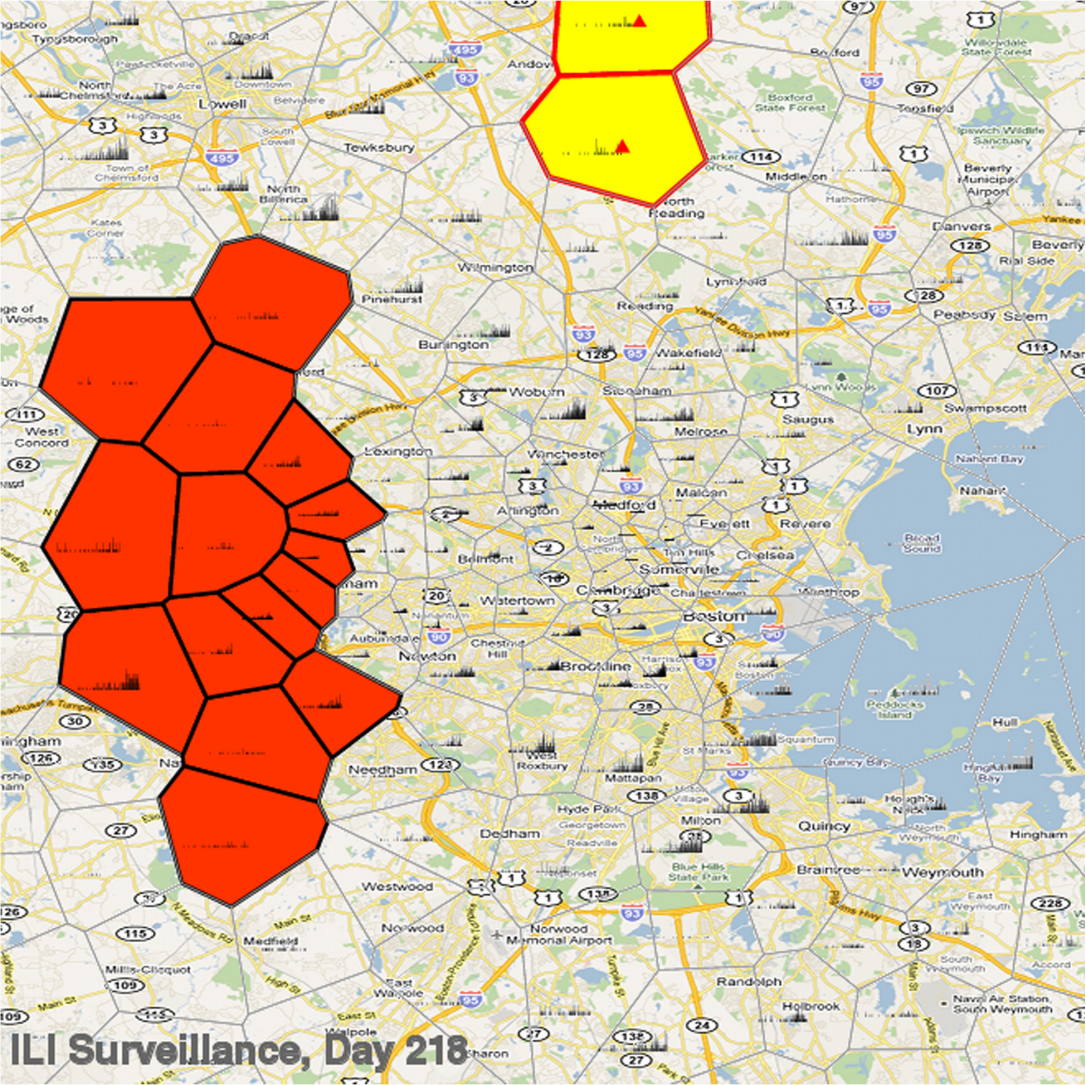
**Simulated ILI surveillance data for downtown Boston as captured by the** “**Natural 6**” **surveillance system.** Surveillance counts per day centered in each zip code location are shown as histograms within each zip code. Detection of an inserted test outbreak (red triangle) is indicated by red bordered zip codes and a false-positive outbreak by black bordered zip codes.

A detailed view of the simulated surveillance data is seen in Figure 3. The zip code boundaries are illustrated as a Voronoi tessellation [23] based on the centroid of the zip code since these are the locations used by the scan-statistic for clustering purposes and not all zip codes are included in the surveillance system. The daily counts of reported cases per zip code are shown as histograms inside the zip code boundaries. The relative contributions of cases from each zip code to the surveillance system can be seen as can the variability between the locations at similar times during the surveillance period. The detection of an inserted outbreak is illustrated with the red highlighted border, whereas the false positive detection of a cluster that doesn’t overlap with an inserted outbreak is shown with a thick black border. The fill color of the clustered locations reveals the decision threshold at which they were detected. Animated movies showing this process over the course of an entire year can be found in the Additional files 2, 3 and 4 (and http://ndssl.vbi.vt.edu/insilicoSurveillance/index.html).

**Figure 4:**
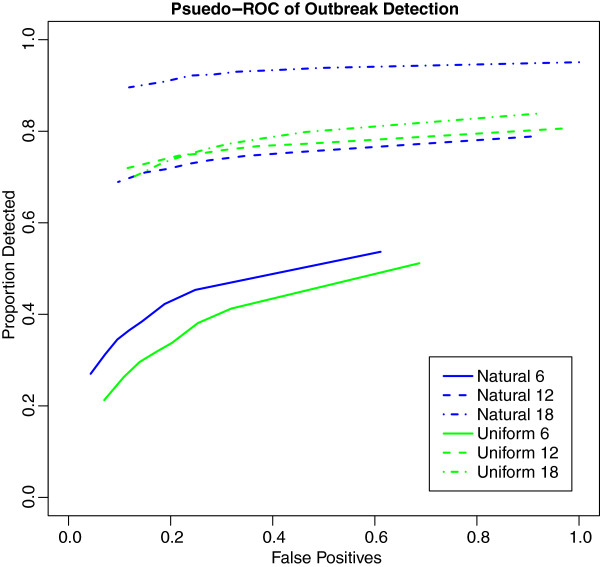
**Pseudo-ROC curves of outbreak detection.** Proportion detected for each surveillance system vs. proportion of all false-positives identified.

Performance of the six surveillance systems is summarized as pseudo receiver operator characteristic curves (pseudo-ROC) [24] in Figure 4. True positives are the proportion of inserted outbreaks detected. False positive proportions used the number of false positives detected at the highest decision threshold among all surveillance systems (Natural 18 with p-value = 0.0333) as the denominator for all surveillance systems. As coverage increases so does the likelihood of detection, though the rate of improvement is non-linear and differs in the two coverage distributions. The decision threshold influences the proportion of false-positives more than the probability of detection, though the effect on probability of detection is stronger in the region of decision thresholds likely to have tenable false positive rates (p-value = 0.005 to 0.001). Across all the surveillance systems, sensitivity improves slightly with increased decision threshold size, however the proportion of false positives increases much more rapidly. Overall, the “Natural” surveillance geography outperforms the “Uniform” surveillance systems.

**Figure 5:**
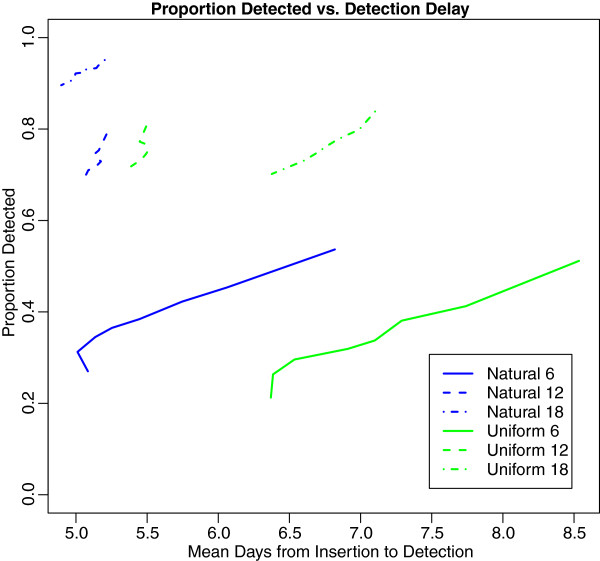
Proportion detected vs. mean days to detection across decision thresholds.

For all detected outbreaks, the delay from insertion to detection can also be measured. The performance of each surveillance system with respect to the mean detection delay and the probability of detection is shown in Figure 5. Given the experimental design, these delays are the theoretical earliest point of detection by a public health official.

The surveillance systems based on the “Natural” surveillance geography consistently show earlier detection than the “Uniform” surveillance geography, even when the probability for detection is essentially the same between the two. Interestingly, higher coverage does not lead to significantly earlier detection: in the case of the “Uniform” surveillance geography, the highest coverage level leads to later detection.

## Conclusions

Agent-based simulation models incorporating important processes that lead to realistic simulated surveillance data is sufficient for evaluating the performance of a surveillance system for outbreak detection. We demonstrated the use of synthetic methods by conducting an experiment to assess how the level of coverage and geographic distribution in a surveillance system affect performance. This kind of experiment can be used to design, as well as to evaluate, surveillance systems.

Description of the study design and the details of its implementation illustrate the flexibility of the methodology. For example, the analysis could be extended to include additional types of inserted outbreaks, and the model could be altered to supply gastrointestinal surveillance. Keeping the scale and level of detail in the model similar to that of the real-world surveillance system facilitates the analysis of the results and makes the simulation results more compatible with existing evaluation tools and methods. These characteristics support the use of agent-based modeling approaches to a wider variety of public health problems.

Our study shows that the geographic distribution of a surveillance system can have a stronger influence on its ability to detect outbreaks than the level of coverage. This and similar studies can also give guidance to making operational decisions, such as selecting a decision threshold for defining a signal that balances an acceptable number of false-positives against a desired probability of outbreak detection.

The 6-fold difference between the nominal false positive rate of 36.5 signals per hundred years at p-value 0.001 (e.g. [365 days * 100 years] * 0.001) and the observed count of 244 signals in the simulated “Natural 6” data with no inserted outbreaks suggests that the spatio-temporal clustering in the realized simulations is indeed quite different from the null hypothesis of case counts proportional to the population. This confirms that the agent-based model has the desired effect on the pattern of cases. It also shows how the process can help public health authorities anticipate the false positive signaling of such systems.

Efforts to enhance existing surveillance systems can be guided by testing different surveillance systems using methods similar to those described here. For example, the impact of adding a new clinic that would draw patients from additional locations in the population could be simulated to determine the value of the additional information. To optimize a surveillance system’s ability to catch outbreaks, a study design that tests the *in silico* surveillance system against a variety of simulated outbreaks and outbreak detection algorithms could be conducted. An assessment of the benefit of finer-grain information could also be conducted: rather than base the outbreak detection on the centroids of the home zip code locations, one could add the zip code location of the school or workplace, or replace zip code centroids with anonymized home addresses.

The high level of detail is a great benefit of agent-based methods; however, it is also produces what is perhaps their biggest complication. With so many parameters, proper calibration can be time consuming and the opportunity for errors and biases can increase. Similarly, it is sometimes difficult to find suitable data for parameterizing high fidelity models of the real world. For such reasons, these methods are not always intended to provide definitive quantitative predictions; rather, they are intended as a tool for comparisons across several designs or outbreak detection methods. Additionally, the methods are computationally intensive and very few health departments have the resources or personnel to perform them. Fortunately advances in scalable high-performance computing and web services have already begun to provide these resources at relatively low cost and with increasing user-friendliness. Work on developing flexible cyber-infrastructures should make it possible to provide a relatively simple web-based interface for users to conduct studies such as this one in the near future.

Further study using these methods is warranted and will include broader investigation into evaluating different outbreak detection algorithms and surveillance systems. These methods will be useful for developing the next generation of outbreak detection tools. With a very large library of simulated background disease, one can use classification techniques and machine learning to develop “proactive” surveillance systems. These would use state assessments to guide public health officials about where to look for further evidence of an outbreak. For instance, an outbreak that might not create a signal for another week could be preceded by weak non-signaling outbreaks in other regions; when taken individually these might be missed, but when considered as a whole this pattern could improve confidence that a significant outbreak is about to occur. This could reduce the burden on PH departments from following up “unlikely” events and improve PH response times to outbreaks by several days. The techniques needed to accomplish this would be very difficult to apply to limited historical data, thus requiring an approach like this one to generate large volumes of plausible high-fidelity data.

Highly detailed simulation models of infectious disease transmission can be configured for many purposes serving public health. We have demonstrated a flexible framework for using such a model for the evaluation and design of surveillance systems and outbreak detection. While there are limitations to the accuracy with which these models can represent the real world, they can provide sufficiently realistic data at a level of detail that enables previously impossible public health research.

## Competing interests

The authors have no competing interests to disclose.

## Authors’ contributions

BL conceived and designed the experiments, carried out the simulations, conducted the experiments, and drafted the manuscript. SE participated in the design of the experiment and in running the simulations. AA participated in the SaTScan analysis. KK participated in the design the experiment, assisted in the analysis of the results, and drafted the manuscript. All authors read, edited, and approved the final manuscript

## Pre-publication history

The pre-publication history for this paper can be accessed here:

http://www.biomedcentral.com/1472-6947/13/12/prepub

## Supplementary Material

Additional file 1 **Detailed Disease Model: A state machine representation of the possible disease states in the simulation.** Starting in the white block on the left an individual can move between different states of susceptibility (representing seasonal effects) over time, once infected they progress through an incubated infected stage into a symptomatic/asymptomatic infectious stage and then into recovered stages. Each state's susceptibility, relative infectiousness, and duration is specified. Click here for file

Additional file 2 **Animation of the Natural surveillance system with 6% coverage detecting, missing, and signaling false positives over the course of a year.** The counts per day per location are visualized as epicurves centered on each location. Locations that are identified as part of a cluster are filled in with a color ranging from red to pale yellow denoting the statistical significance of the identified cluster (red = p-value of 0.001 and pale yellow = p-value of 0.03333). Inserted artificial outbreaks are shown as red triangles in the epicurves and the locations are also outlined in red. False-positive outbreaks are outlined with bold borders in black. Click here for file

Additional file 3 **Animation of the Natural surveillance system with 18% coverage detecting, missing, and signaling false positives over the course of a year.** The counts per day per location are visualized as epicurves centered on each location. Locations that are identified as part of a cluster are filled in with a color ranging from red to pale yellow denoting the statistical significance of the identified cluster (red = p-value of 0.001 and pale yellow = p-value of 0.03333). Inserted artificial outbreaks are shown as red triangles in the epicurves and the locations are also outlined in red. False-positive outbreaks are outlined with bold borders in black. Click here for file

Additional file 4 **Animation of the “Uniform” surveillance system with 6% coverage detecting, missing, and signaling false positives over the course of a year.** The counts per day per location are visualized as epicurves centered on each location. Locations that are identified as part of a cluster are filled in with a color ranging from red to pale yellow denoting the statistical significance of the identified cluster (red = p-value of 0.001 and pale yellow = p-value of 0.03333). Inserted artificial outbreaks are shown as red triangles in the epicurves and the locations are also outlined in red. False-positive outbreaks are outlined with bold borders in black. Click here for file
