# Supplementary figures and images for: in silico Surveillance: evaluating outbreak detection with simulation models
Source: BMC Med Inform Decis Mak. 2013 Jan 23;13:12. doi: 10.1186/1472-6947-13-12 (PMC3691709; doi:10.1186/1472-6947-13-12)

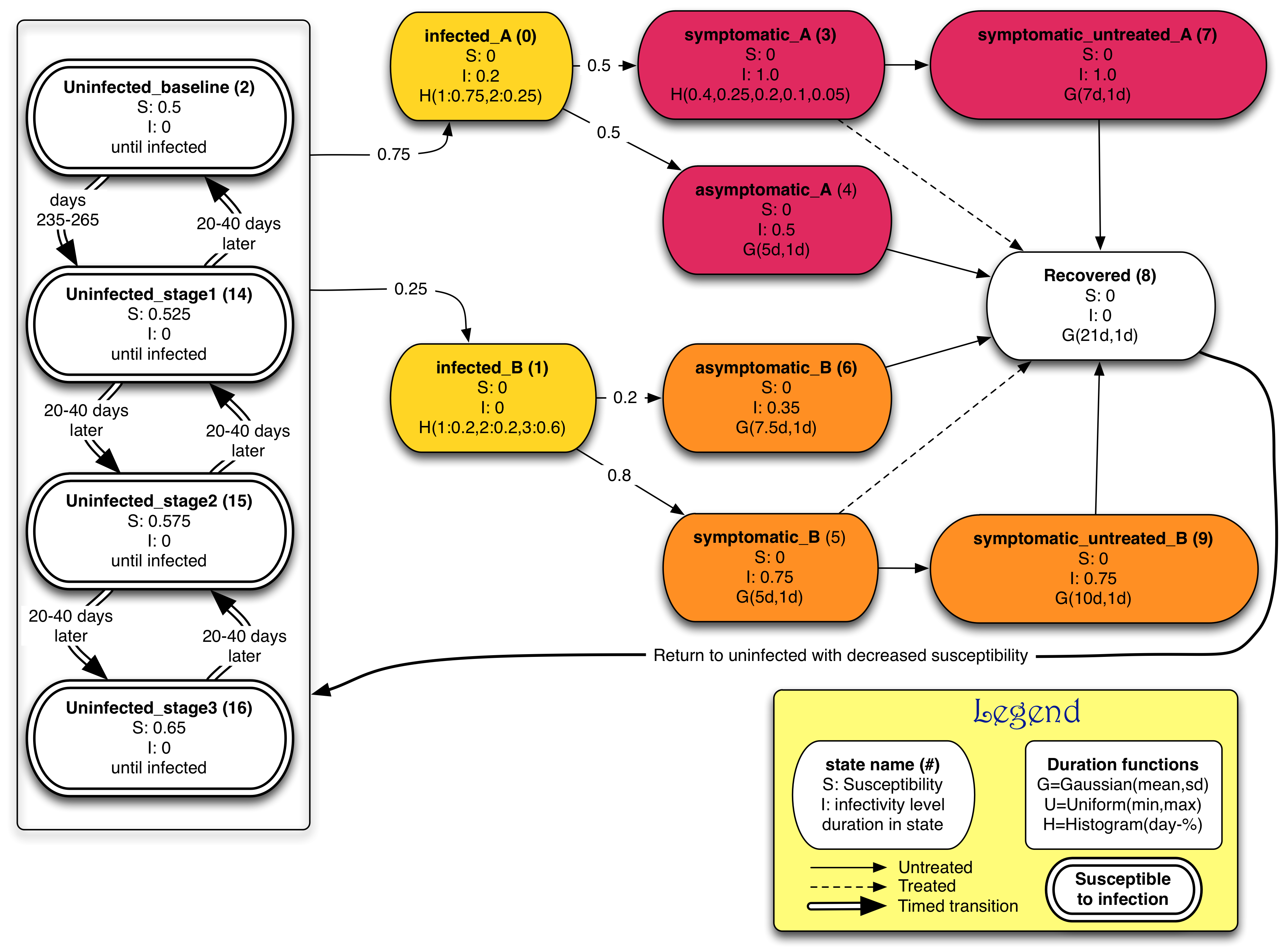

Supplement: Additional file 1 — Detailed Disease Model: A state machine representation of the possible disease states in the simulation. Starting in the white block on the left an individual can move between different states of susceptibility (representing seasonal effects) over time, once infected they progress through an incubated infected stage into a symptomatic/asymptomatic infectious stage and then into recovered stages. Each state's susceptibility, relative infectiousness, and duration is specified. [file 1472-6947-13-12-S1.png]
